# Active Transport of Lignin Precursors into Membrane Vesicles from Lignifying Tissues of Bamboo

**DOI:** 10.3390/plants10112237

**Published:** 2021-10-20

**Authors:** Natsumi Shimada, Noriaki Munekata, Taku Tsuyama, Yasuyuki Matsushita, Kazuhiko Fukushima, Yoshio Kijidani, Keiji Takabe, Kazufumi Yazaki, Ichiro Kamei

**Affiliations:** 1 Graduate School of Agriculture, University of Miyazaki, Miyazaki 889-2192, Japan; gb14025@student.miyazaki-u.ac.jp (N.S.); gb16043@student.miyazaki-u.ac.jp (N.M.); kijiyo@cc.miyazaki-u.ac.jp (Y.K.); kamei@cc.miyazaki-u.ac.jp (I.K.); 2 Graduate School of Bioagricultural Sciences, Nagoya University, Nagoya 464-8601, Japan; fx7789@go.tuat.ac.jp (Y.M.); kazu@agr.nagoya-u.ac.jp (K.F.); 3 Graduate School of Agriculture, Kyoto University, Kyoto 606-8502, Japan; takabe.keiji.52w@st.kyoto-u.ac.jp; 4 Research Institute for Sustainable Humanosphere, Kyoto University, Uji 611-0011, Japan; yazaki@rish.kyoto-u.ac.jp

**Keywords:** membrane transport, lignification, *Phyllostachys pubescens*, coniferin, *p*-glucocoumaryl alcohol, secondary active transport

## Abstract

Lignin is the second most abundant natural polymer on Earth and is a major cell wall component in vascular plants. Lignin biosynthesis has three stages: biosynthesis, transport, and polymerization of its precursors. However, there is limited knowledge on lignin precursor transport, especially in monocots. In the present study, we aimed to elucidate the transport mode of lignin monomers in the lignifying tissues of bamboo (*Phyllostachys pubescens*). The growth manners and lignification processes of bamboo shoots were elucidated, which enabled us to obtain the lignifying tissues reproducibly. Microsomal membrane fractions were prepared from tissues undergoing vigorous lignification to analyze the transport activities of lignin precursors in order to show the ATP-dependent transport of coniferin and *p*-glucocoumaryl alcohol. The transport activities for both precursors depend on vacuolar type H^+^-ATPase and a H^+^ gradient across the membrane, suggesting that the electrochemical potential is the driving force of the transport of both substrates. These findings are similar to the transport properties of these lignin precursors in the differentiating xylem of poplar and Japanese cypress. Our findings suggest that transport of coniferin and *p*-glucocoumaryl alcohol is mediated by secondary active transporters energized partly by the vacuolar type H^+^-ATPase, which is common in lignifying tissues. The loading of these lignin precursors into endomembrane compartments may contribute to lignification in vascular plants.

## 1. Introduction

Lignin is a major cell wall component of vascular plants and the second most abundant natural polymer on Earth. Lignin contributes to efficient water transport in xylem tissues, plant posture maintenance, and microorganism resistance in vascular plants. Lignin predominantly comprises *p*-hydroxyphenyl (H), guaiacyl (G), and syringyl (S) units, depending on the biosynthetic pathway. Lignin biosynthesis consists of three steps: the biosynthesis of lignin precursors (lignin monomers) in the cell, transport of the precursors from the cytosol to the cell wall, and polymerization in the cell wall. The biosynthesis and polymerization of lignin precursors have been intensively studied, whereas knowledge on the transport of lignin precursors is limited [1,2,3].

To study the transport of lignin precursors, Miao and Liu conducted transport experiments using microsomal membrane fractions derived from rosette leaves of *Arabidopsis thaliana* [4]. Microsomal vesicles obtained from rosette leaves revealed the ATP-dependent transport activities of monolignols, coniferyl alcohol, and sinapyl alcohol, as well as monolignol glucosides, coniferin, and syringin. Their study suggested that these transport activities are mediated by ATP-binding cassette (ABC)-like transporters. However, the rosette leaves of herbal plants like Arabidopsis have very little lignified tissue, and the parenchyma of the leaves may be involved in these transport activities. One ABC transporter in *A. thaliana*, AtABCG29, was reported to be a transporter of *p*-coumaryl alcohol, the monolignol of the H-unit lignin [5], although the amount of H-unit lignin is very limited in most vascular plants. Studies on *A. thaliana* have attempted to identify the ABC transporters of lignin precursors [6,7,8]; however, the transporters of lignin precursors of the G and S units have not been elucidated.

While the transporters may be involved in the translocation of lignin precursors from the cytosol to the cell wall, it is also possible that the lignin precursors, such as monolignols, can move across membranes via passive diffusion because they are small, relatively hydrophobic molecules. A model experiment with a lipid bilayer disk showed that the phenolic compounds were partitioned into the lipid bilayer disks without transporter proteins [9]. Additionally, computational simulations predicted that most lignin-related compounds, including monolignols, can readily permeate across model biological membranes. However, glycosylated or carboxylated lignin precursors revealed low levels of membrane permeability due to their hydrophilic properties, indicating that the translocation of hydrophilic precursors across membranes needs transporters or transport machinery with carrier proteins [10].

Monolignol glucosides, i.e., *p*-glucocoumaryl alcohol, coniferin, and syringin, have been considered the storage forms of lignin precursors and the forms to be transported toward outside cells [11]. In particular, coniferin is present in the differentiating xylem of conifers and appears to be relevant for the G-unit of lignin. The coniferin content in the differentiating xylem is highest during the early stage of xylem formation and decreases with the progress of secondary cell wall formation in *Pinus thunbergii* [12], Japanese cypress (*Chamaecyparis obtusa*) [13], and *Ginkgo biloba* [14]. Raman spectroscopy and X-ray CT analyses have demonstrated that coniferin exists in the lumen of the tracheid cells during the formation of the S_1_ (the outer layer of the secondary wall) and S_2_ (the middle layer of the secondary wall) layers; however, coniferin disappears in the cells during the formation of the S_3_ layer (the inner layer of the secondary wall) and during the lignification stage [15,16]. Furthermore, the administration of radiolabeled monolignol glucosides results in the incorporation of labeling into the cell walls of differentiating xylems [17,18,19]. Thus, coniferin is not a final product to be accumulated but is actively utilized for the lignification of secondary cell walls.

Biochemical experiments using microsomal membrane vesicles prepared from the actively lignifying tissues of woody plants suggest that vacuolar type H^+^-ATPase (V-ATPase) and the H^+^ gradient are involved in coniferin transport, which are common in both poplar (*Populus sieboldii* × *Populus grandidentata*) and Japanese cypress [16]. A similar ATP-dependent transport activity with the same biochemical properties was also observed in another monolignol glucoside involved in H-unit of lignin, *p*-glucocoumaryl alcohol [20]. Recently, almost identical transport activities have been found in the differentiating xylems of Norway spruce and tobacco cultured cells [21]. These studies were performed using conifers, broad-leaved trees, and *A. thaliana*, but little is known about the transport of lignin precursors in monocots.

In the context of biomass production for a sustainable society, bamboo has been intensively studied, owing to its fast growth in recent years. The elucidation of lignin biosynthesis mechanisms in bamboo allows for the regulation of lignin quality and quantity, leading to efficient biomass utilization. While lignin biosynthesis in monocots has been actively studied [22,23,24,25,26], the transport of lignin precursors has not been investigated to date.

In the present study, we examined the transport activities of lignin monomers necessary for the lignifying tissues of bamboo. The transport activities of coniferin and *p*-glucocoumaryl alcohol were identified, and it was found that their transport modes were similar to those of poplar and Japanese cypress, suggesting that the active transport of monolignol glucosides into intracellular compartments is conserved among lignifying tissues and commonly contributes to lignification in vascular plants.

## 2. Results and Discussion

### 2.1. Elongation Growth and Lignification Processes in Bamboo Shoots

The growth rate of bamboo shoots is rapid, so predicting the growth and lignification stages of each culm or each internode is difficult, especially at the early growth stages. Therefore, the elongation growth and lignification processes of bamboo shoots were elucidated to identify the lignification stage of each internode and to obtain the lignifying tissues quickly at the harvesting site.

**Figure 1:**
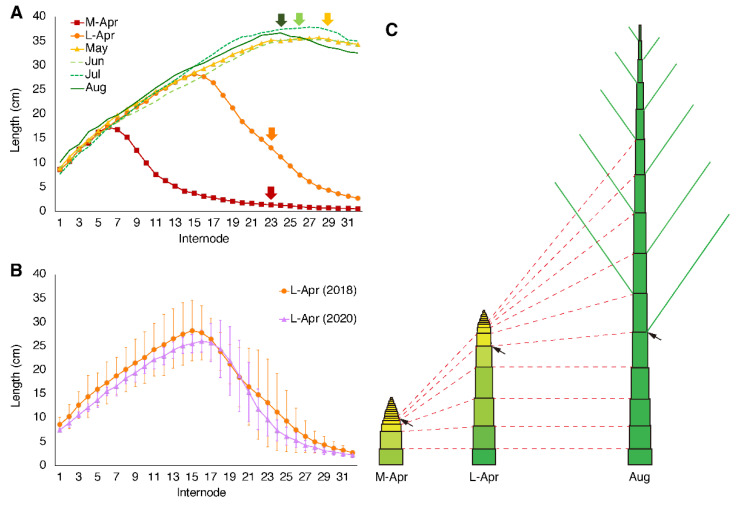
Elongation pattern of moso bamboo shoots. (**A**,**B**), Lengths of internodes (up to the 32nd internode) of bamboo shoots collected during different seasons ((**A**), see Table 1) or in the same season of different years (**B**). (**C**), A simple diagram of the elongation of the internodes. The internodes at the base of the sample or the uppermost internodes from which the roots grew were designated as internode ‘0’, and the internode numbers were assigned sequentially from the ground to the top. Arrows indicate the lowest node at which a lateral bud appeared ((**A**,**C**); July samples had no lateral bud data). Data are means of three biological replicates. Error bars represent SD (**B**).

The bamboo shoots we used in this study were *Phyllostachys pubescens*, which began growing in March in Nobeoka, Japan. The elongation of bamboo shoots was observed until mid-May, when they reached lengths of approximately 16 m, which was consistent with previous reports [27]. At the early developmental stages, the lower internode lengths were longer than upper internodes, which was similar to results previously reported for several bamboo species [27,28]. The internode of the nearest basement was set to internode 0, and each internode was numbered sequentially from the lowest to the highest. With development completed, around the 26th internode was the longest on the culm (Figure 1A). The length of the longest internode was approximately 40 cm, which is typical for *P. pubescens* [27]. The lengths of each internode were similar in culms after the completion of elongation (Figure 1A) or in culms at the same growth stage in different years (Figure 1B). These results indicated that elongation of internodes was completed successively from the internodes near the basement (Figure 1C). Furthermore, the lowest lateral buds appeared around the 26th node among the shoots (Figure 1A, arrows). The lengths of the internodes and the branching manner may be determined during the early stages of differentiation.

**Figure 2:**
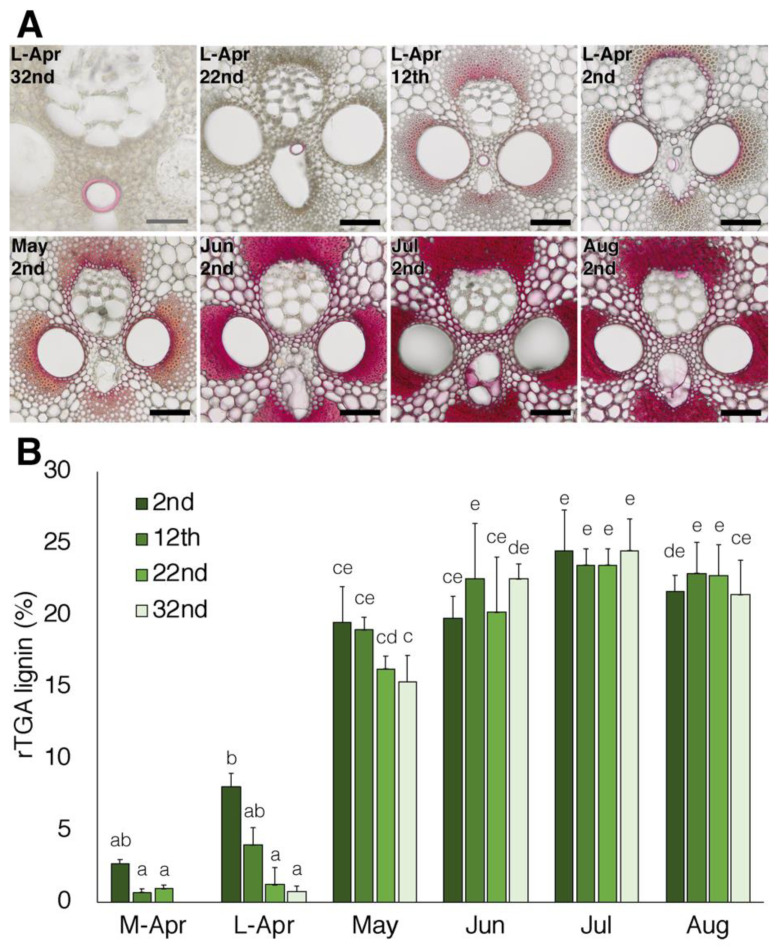
Lignin distribution in developing culms of moso bamboo. (**A**), Histochemical analysis of culm cell walls. Cross sections of culms were stained by phloroglucinol-HCl reagents for lignin detection. Bars = 100 µm except the L-Apr 32nd sample (gray, 50 µm). (**B**), Lignin content was determined using the rapid thioglycolic acid (rTGA) method. M-Apr; middle of April, L-Apr; late of April. See Table 1 for sample details. Data are the means of three biological replicates. Error bars = SD. Different letters indicate significant differences (*p* < 0.05, Tukey’s test).

The lignin distribution in the growing bamboo culms was examined by Wiesner reaction and the lignin content in each internode was determined using the rapid thioglycolic acid method (Figure 2). Lignification occurred first in the protoxylem, then in the metaxylem, vascular fiber, and finally in the parenchyma of the ground tissue (Figure 2A). The lignin content increased from the lower internodes toward the tip of the bamboo shoots during the early growth stages. The lignin content increased gradually until July, even after the cessation of elongation growth, and reached about 25% in every internode (Figure 2B). These results are in a similar range to those previously published for shoots of *P. pubescens* [29] and *Sinobambusa tootsik* [28]. Thus, the growth and lignification processes may be similar in different bamboo species.

### 2.2. Transport Activities of Lignin Monomers in the Lignifying Tissues of Bamboo

**Figure 3:**
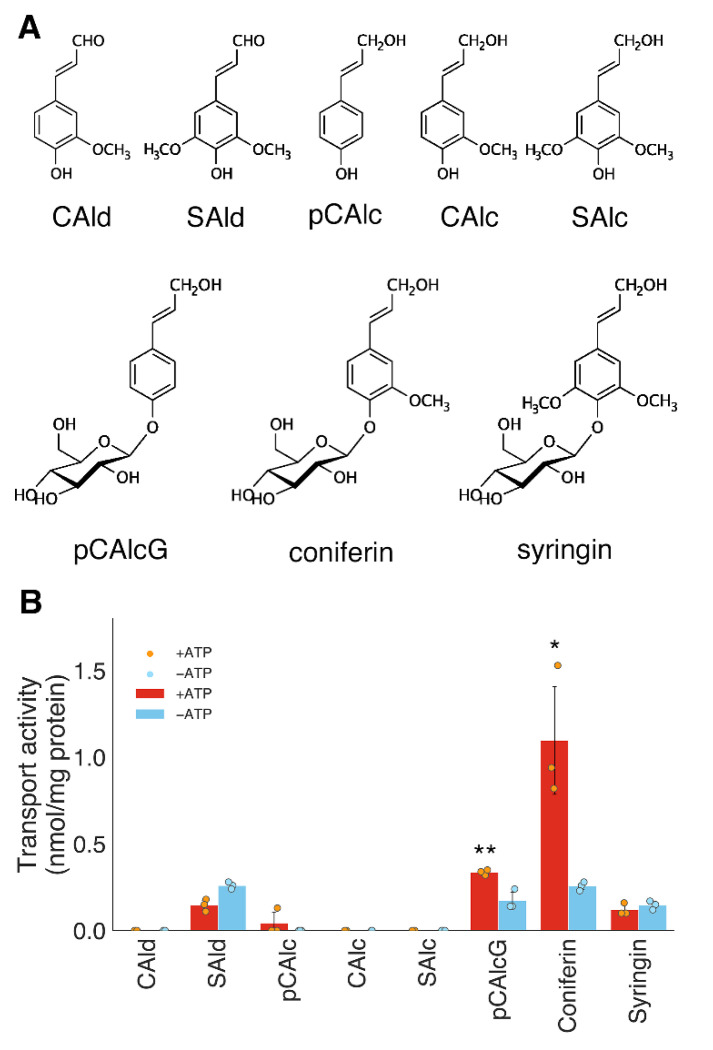
Transport activities of lignin precursors in membrane vesicles prepared from shoots of moso bamboo. (**A**), Chemical structures of lignin precursors used in the present study. CAld; coniferaldehyde, SAld; sinapaldehyde, pCAlc; *p*-coumaryl alcohol, SAlc; sinapyl alcohol, pCAlcG; *p*-glucocoumaryl alcohol; (**B**), Substrate specificity of uptake activity into the microsomal membrane vesicles. Membrane vesicles were obtained from the 10th internodes of mid-April (M-Apr) samples of moso bamboo. Data are the means of three replicates (error bars = SD). * *p* < 0.05, ** *p* < 0.01 (student’s *t*-test).

Few studies have investigated the transport activities of lignin precursors, especially in monocot. In the present study, we detected the active transport of lignin monomers in the lignifying tissues of bamboo. Internode tissues actively undergoing lignification were selected according to the data of Figure 1 and Figure 2, and microsomal membrane fractions were prepared from these tissues. Using the membrane fractions biochemical transport experiments were performed, in which lignin precursors were used as transport substrates. The substrates tested were aglycones of lignin monomers, i.e., coniferaldehyde, sinapaldehyde, *p*-coumaryl alcohol, coniferyl alcohol, and sinapyl alcohol, along with some of their glucosides, *p*-glucocoumaryl alcohol, coniferin, and syringin (Figure 3A). As a result, we demonstrated clear ATP-dependent transport activities of coniferin and *p*-glucocoumaryl alcohol in the microsomal membrane vesicles prepared from the bamboo shoots during the early lignification stage (Figure 3B). Such specificity of transported lignin precursors is identical with poplar, Japanese cypress, and Norway spruce [16,20,21].

No transport activity was detected in aglycones of monolignol or cinnamaldehyde (Figure 3B) across microsomal membrane vesicles containing plasma membranes with H^+^-ATPase activities (Figure S1). These lignin precursors are supposed to move across membranes by passive diffusion due to their hydrophobic properties [10]. The plasticity of lignin precursors incorporated into lignin polymers has been observed in various mutants [2], implying that passive diffusion or less-specific transport is involved in lignification. Passive diffusion of monolignols refers to movement across the lipid bilayer and does not require energy or specific transporters; however, its flux is difficult to control by changing the cytosolic concentration of monolignols because some monolignols are toxic to plant cells [5,30]. The transport modes of lignin precursors may depend on cell or tissue types as lignification patterns vary according to cell or tissue types [31]. In lignifying tissues in particular, no passive transport of monolignols or cinnamaldehyde was observed in the present study (Figure 3B) or in previous biochemical studies [16,20,21]. Detailed examinations are needed to investigate the possible passive diffusion for monolignol or cinnamaldehyde across biological membranes.

**Figure 4:**
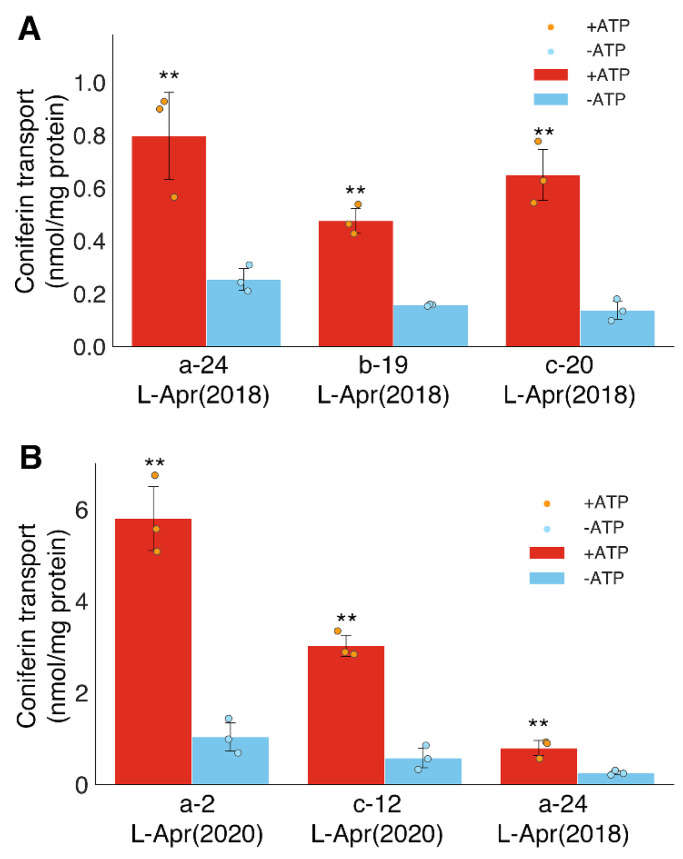
Transport activity of coniferin in different internodes in different culms of moso bamboo. (**A**), Coniferin transport activity in different culms collected in late April (L-Apr (2018)). a-24, the 24th internode of the culm a; b-19, the 19th internode of the culm b; c-20, the 20th internode of the culm c; (**B**), Coniferin transport activity in different internodes collected in late April. a-2, the 2nd internode of the culm a collected in L-Apr (2020); c-12, the 12th internode of the culm c collected in L-Apr (2020); a-24, the 24th internode of the culm a collected in L-Apr (2018). Data are the means of three technical replicates (error bars = SD). ** *p* < 0.01 compared with/without ATP (student’s *t*-test).

The ATP-dependent transport activity of coniferin was demonstrated in all the microsomal fractions obtained from different bamboo shoots (Figure 4), which suggests that the developing tissues of *P. pubescens* generally possess coniferin transport activity. Microsomal membrane vesicles were obtained from different internodes (the 2nd, 12th, and 24th internode) of late April samples at different lignification stages (Figure 2). Transport assays clearly showed that all microsomal membranes possess coniferin transport activities (Figure 4B). The transport activity of coniferin in membrane vesicles obtained from the 2nd internode was higher than those from the 24th internode, which is consistent with the lignification stages; namely, in late April samples, internodes around the 22nd internode were under the early lignification stage, whereas lignification was proceeding vigorously in the 2nd internode (Figure 2). These results support the idea that the coniferin transport found in the present study is involved in the lignification of bamboo shoots.

### 2.3. Transport Mode of Coniferin in Bamboo Shoots

**Figure 5:**
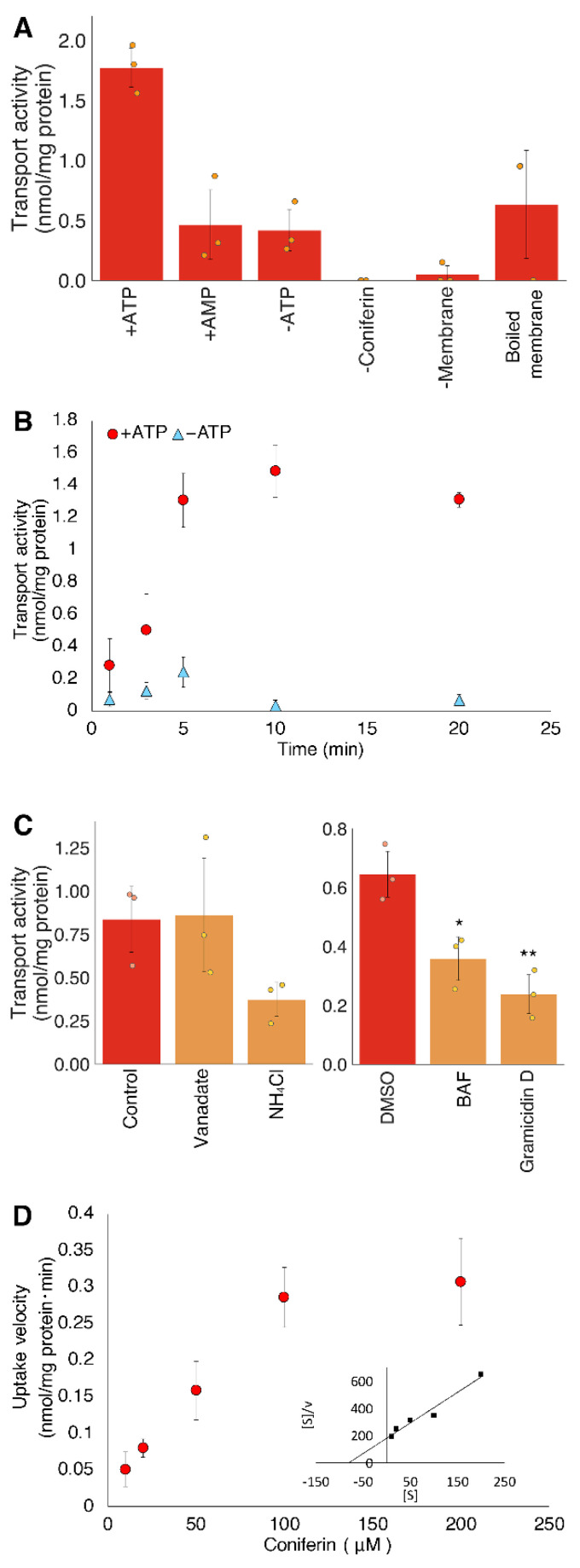
Biochemical characterization of transport activity of coniferin. (**A**), Negative controls for coniferin uptake. Membrane vesicles were incubated with 100 μM coniferin either with 5 mM Mg/ATP, with 5 mM Mg/AMP (+AMP), or without Mg/ATP (−ATP). “Boiled membrane” refers to coniferin uptake by use of heat-denatured microsomal fraction; (**B**), Time course of coniferin uptake. Membrane vesicles were incubated with 100 μM coniferin in the presence (●) or absence (○) of 5 mM Mg/ATP; (**C**), Inhibitor experiments of coniferin transport. Microsomal membranes were incubated with 100 μM coniferin and 5 mM Mg/ATP, to which vanadate (1 mM), NH_4_Cl (10 mM), bafilomycin A1 (BAF) (1 μM) and gramicidin D (25 μM) were added independently; (**D**), Membrane vesicles were incubated for 5 min in the presence of 5 mM Mg/ATP and each coniferin concentration. Inset shows Hanes–Woolf plots. Data are the means of three technical replicates (error bars = SD). * *p* < 0.05, ** *p* < 0.01 compared with DMSO (Dunnett test).

Further transport experiments were conducted to characterize the coniferin transport in the lignifying tissues of bamboo shoots. A decrease in the coniferin transport activity was observed when AMP was used instead of ATP. In addition, heat-denatured microsomal membrane fractions showed reduced transport activity (Figure 5A). This suggests that the active transport energized by ATP hydrolysis is involved in the membrane transport of coniferin.

Our time-course experiment showed that the coniferin transport activity increased rapidly after 10 min in the presence of ATP (Figure 5B). Under conditions lacking ATP, no transport activity was observed even after 20 min. Thus, the active transport of coniferin appears to be mediated by a transporter and the passive diffusion hardly occurs in the microsomal membranes for this hydrophilic transport substrate.

To elucidate the mode of coniferin transport, various inhibitors were tested in transport assays (Figure 5C). Coniferin transport was not inhibited by vanadate, a typical inhibitor of ABC transporters and P-type ATPase localized at the plasma membrane, but it was inhibited by bafilomycin A1, an inhibitor of V-ATPase. The inhibition of coniferin transport by the H^+^-gradient erasers NH_4_Cl and gramicidin D indicates that V-ATPase and H^+^ gradient are involved in coniferin transport in the lignifying tissues of bamboo. These findings suggest that coniferin transport is mediated by a secondary transporter that is energized partly by V-ATPase. This coniferin transport mode in bamboo is similar to the differentiating xylems in poplar and Japanese cypress [16], implying that the active transport of coniferin driven by an electrochemical gradient is conserved in lignifying tissues of coniferous, dicot, and monocot plants.

We also tried to fractionate microsomal membranes to examine what kind of membranes are involved in the coniferin transport in moso bamboo; however, the membrane could not be fractionated clearly (Figure S2). Still, inhibition of the coniferin transport not by vanadate, an inhibitor of P-type ATPase localized at the plasma membrane, but by bafilomycin A1, an inhibitor of V-ATPase localized at the endomembrane system, indicates that the endomembrane, rather than the plasma membrane, is involved in coniferin transport in bamboo shoots. V-ATPase is associated with not only tonoplast, but also the ER membrane, Golgi membrane, and other membranes of small vesicles [32]. It is hypothesized that coniferin is transported into V-ATPase-associated endomembrane compartments, which are exocytosed to release coniferin into the cell wall [16,33].

When the microsomal membrane fractions were incubated with different concentrations of coniferin, the uptake rate increased along with the substrate concentration according to Michaelis–Menten-type kinetics (Figure 5D). The apparent *K*_m_ value of the coniferin transport was calculated using a Hanes–Woolf plot as being 32–87 µM, which is similar to that of coniferin transport in poplar and Japanese cypress [16]. This result corroborates that homologous transporters for coniferin transport are conserved in the lignified tissues of various vascular plants.

Previous studies using microsomal membrane fractions from *A. thaliana* leaves have suggested that ABC transporters mediate the translocation of coniferin and syringin in vacuolar membrane vesicles [4]. However, the transport of coniferin observed in the present study suggests the involvement of a coniferin/H^+^ antiporter, not an ABC transporter, as indicated in the differentiating xylem of woody plants [16]. *A. thaliana* rosette leaves contain a large portion of mesophyll cells but have little lignifying tissue, while the bamboo shoots used in the present study largely consist of lignifying tissues. The transport modes observed in the rosette leaves of herbal plants are different from those in the lignifying tissues of bamboo and woody plants as these tissues play definitively different roles.

### 2.4. Transport of p-Glucocoumaryl Alcohol in Bamboo Shoots

**Figure 6:**
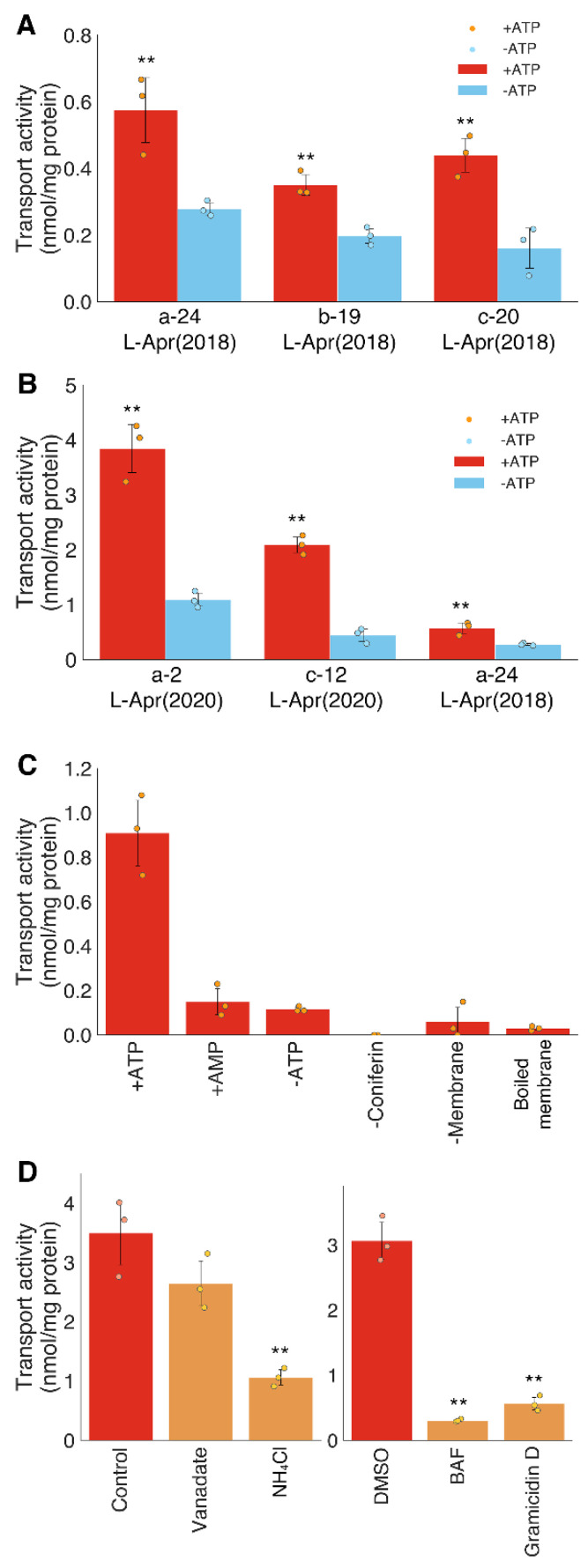
Transport activity of *p*-glucocoumaryl alcohol by membrane vesicles of *P. pubescens*. (**A**), *p*-Glucocoumaryl alcohol transport activity in different culms collected in late April (L-Apr (2018)). a-24, the 24th internode of the culm a; b-19, the 19th internode of the culm b; c-20, the 20th internode of the culm c; (**B**), *p*-Glucocoumaryl alcohol transport activity in different internodes collected in late April. a-2, the 2nd internode of the culm a collected in L-Apr (2020); c-12, the 12th internode of the culm c collected in L-Apr (2020); a-24, the 24th internode of the culm a collected in L-Apr (2018); (**C**), Negative controls for uptake activity of *p*-glucocoumaryl alcohol. Microsomal membrane vesicles were incubated in 100 μM *p*-glucocoumaryl alcohol with 5 mM Mg/ATP, with 5 mM Mg/AMP (+AMP), or without Mg/ATP (−ATP). “Boiled membrane” refers to the heat-denatured microsomal fraction; (**D**), Microsomal membrane vesicles were incubated with 100 μM *p*-glucocoumaryl alcohol and 5 mM Mg/ATP, to which vanadate (1 mM), NH_4_Cl (10 mM), bafilomycin A1 (BAF, 1 μM) and gramicidin D (25 μM)were added independently. Data are the means of three technical replicates (error bars = SD). ** *p* < 0.01 compared with/without ATP by student’s *t*-test (**A**,**B**) or compared with control or DMSO by Dunnett test (**D**).

Transport experiments were further conducted to characterize the transport activity of another monolignol glucoside, *p*-glucocoumaryl alcohol, using the lignifying tissues of bamboo shoots (Figure 6A,B). This compound is especially important for the formation of H-unit lignin. The transport activity of *p*-glucocoumaryl alcohol was abolished by the addition of AMP instead of ATP; additionally, heat denatured membrane vesicles did not show clear transport activity, even in the presence of ATP (Figure 6C). Thus, the active transport of *p*-glucocoumaryl alcohol may, like that of coniferin, consume energy obtained from ATP hydrolysis.

Transport assays using various inhibitors showed that the transport activity of *p*-glucocoumaryl alcohol was significantly inhibited by a V-ATPase inhibitor (bafilomycin A1) and by H^+^ gradient erasers (NH_4_Cl and gramicidin D), but it was not inhibited by an ABC transporter inhibitor (vanadate) (Figure 6D). Thus, an electrochemical gradient created partly by V-ATPase appears to be involved in the transport of *p*-glucocoumaryl alcohol, as well as coniferin. Recently, the ATP-dependent transport activity of *p*-glucocoumaryl alcohol has been detected in the differentiating xylems of several species, and similar characteristics for the transport activity have been reported [20,21]. *p*-Glucocoumaryl alcohol transport mediated by electrochemical gradient and V-ATPase may also be conserved in the lignifying tissues of coniferous, dicot, and monocot plants.

The incorporation of radiolabeled *p*-glucocoumaryl alcohol was shown in differentiating xylems of not only a gymnosperm [17], but also magnolia, beech, lilac, and poplar [18]. Furthermore, *p*-glucocoumaryl alcohol has been detected in developing bamboo, and its content peaks during the early stage of lignification [28]. These results imply that *p*-glucocoumaryl alcohol is used for lignification in bamboo; consequently, the *p*-glucocoumaryl alcohol transport identified in the present study may be involved in the lignification of bamboo.

### 2.5. The Role of Monolignol Glucoside Transport in Lignification

Numerous glucosides of phenolic compounds have been investigated and recognized as vacuolar storage substances in many plant species [34,35]. However, coniferin is not considered a dead-end product. Coniferin has often been detected in gymnosperms and its content in the differentiating xylems of gymnosperms peaks at around the cambium and decreases as lignification progresses [12,13]. In addition, coniferin *β*-glucosidase responsible for the delivery of its aglycone, coniferyl alcohol, localize in the cell walls of *Pinus contorta* var. *latifolia* [36] and poplar [33]. Coniferin *β*-glucosidase of rice is also indicated to localize in the cell wall [37]. The transport activities found in the present study may be involved not only in coniferin accumulation in vacuoles but also in coniferin loading into small vesicles, through which coniferin is secreted to the cell wall via membrane dynamics such as exocytosis [16,33].

At present, the roles of monolignol glucosides in lignification are still unclear in dicots, despite several reverse genetic studies. The downregulation of coniferin *β*-glucosidase in an *A. thaliana* mutant resulted in coniferin accumulation, but the lignification did not change [38]. The overexpression of a glycosyltransferase involved in monolignol glucoside biosynthesis in *A. thaliana* resulted in ectopic lignification [39]. However, the overexpression of the UDP-glycosyltransferase involved in monolignol glycosylation in poplar did not affect lignification [40]. Although the roles of monolignol glucosides in lignification are obscure, the present study, in addition to previous studies [16,20,21], clearly shows that the transport activities of coniferin and *p*-glucocoumaryl alcohol are highly conserved in lignifying tissues, implying the importance of the transport of monolignol glucosides in lignification.

Our previous study on bamboo shoots suggested that the contents of monolignol glucoside and free monolignols peak during the early and late lignification stages, respectively [28]. The transport activities may differ depending on the growth stage. However, in the present study we did not obtain microsomal membrane fractions with H^+^-ATPase activities from August samples (Figure S1) that were highly lignified (Figure 2). It is necessary to identify transporters of lignin precursors using genetic and/or molecular approaches to clarify the overall scheme of transport in lignified tissues at the molecular level. Further studies are required to elucidate all the lignification mechanisms of bamboo.

## 3. Conclusions

We first elucidated the precise growth and lignification patterns of elongating bamboo shoots to identify the stage at which tissues are actively lignifying. Microsomal membrane fractions were prepared from bamboo shoots undergoing vigorous lignification to elucidate the transport activities. Biochemical transport assays using eight lignin precursors, both aglycones and glucosides, revealed that coniferin and *p*-glucocoumaryl alcohol were actively transported into microsomal membrane vesicles in an ATP-dependent manner. Transport assays using various inhibitors suggested that the transport of both coniferin and *p*-glucocoumaryl alcohol was mediated by secondary active transporters that depend on the proton motive force generated by the proton pumps like V-ATPase. Such a transport mode is similar to that found in the differentiating xylems of poplar and Japanese cypress. This implies that secondary transporters of coniferin and *p*-glucocoumaryl alcohol are conserved in the lignifying tissues and that the loading of these lignin monomers into V-ATPase associated endomembrane compartments contributes to lignification in vascular plants.

## 4. Materials and Methods

### 4.1. Chemicals

The chemicals used in this study were purchased from Nacalai Tesque (Kyoto, Japan) and Wako Pure Chemicals (Osaka, Japan). Coniferin and syringin were provided by Dr. Noritsugu Terashima of Nagoya University. *p*-Coumaryl alcohol and its glucoside were synthesized as described previously [28,41].

### 4.2. Plant Materials

Shoots of *P. pubescens* were collected from Nobeoka (32°58′58′′ N 131°58′71′′ E), Miyazaki Prefecture, Japan. Three independent culms (a–c) of similar length were collected on each sampling day (Table 1). The internodes at the base of the sample or the uppermost internodes from which the roots grew were designated as internode ‘0′, and the internode numbers were then assigned sequentially from the ground to the top. The total lengths of culms, as well as the length of each internode, were measured (Table 1). Small blocks were prepared from the top of the 2nd, 12th, 22nd, and 32nd internodes and stored at −80 °C. For preparation of microsomal membranes, internodes other than the above were also obtained and frozen in liquid nitrogen followed by storage at −80 °C.

### 4.3. Histochemical Analysis

Each bamboo sample was fixed in 2.5% glutaraldehyde in 0.1 M phosphate buffer (pH 7.2). After fixation, the blocks were washed with water and transverse sections (40-μm thickness) were obtained. The sections were incubated in 2% (*w*/*v*) phloroglucinol in 95% ethanol for 1 min, followed by the addition of 6 M HCl. The incubated sections were observed under a light microscope (DP25, Olympus, Tokyo, Japan).

### 4.4. Chemical Analysis

The lignin content was determined using the rapid thioglycolic acid method [42]. Briefly, approximately 20 mg of the defatted sample, 1 mL of 3 M HCl, and 0.1 mL of thioglycolic acid were heat-treated at 80 °C for 3 h. After centrifugation (20,000× *g*, 10 min), 800 µL of the supernatant was discarded and 1 mL of distilled water was added as a wash. After centrifugation, the supernatant was discarded, 1 mL of 1 M NaOH was added, and the mixture was vertically shaken (120 rpm) for 1 h. After centrifugation (20,000× *g*, 10 min), 1 mL of the supernatant was transferred to a new 1.5-mL tube. Approximately 220 µL of concentrated HCl was added and mixed by inversion. After centrifugation (20,000× *g*, 20 min), the supernatant was discarded, and 1 mL of 1 M NaOH was added to dissolve the pellet. The lignin concentration was determined by measuring the absorbance at 280 nm using a spectrophotometer (MULTISKAN GO; Thermo, Rockford, IL, USA) with a calibration curve prepared with the milled wood lignin of bamboo [43]. The samples were appropriately diluted with 1 M NaOH to measure the absorbance at 280 nm in the range of the calibration curve.

### 4.5. Preparation of Microsomal Membrane Fractions

The following operations were all performed at 4 °C or on ice. Small bamboo blocks without the epidermis were added to a homogenizing KOH buffer (pH 8.0), which contained 100 mM HEPES, 5 mM EDTA, 10% (*v*/*v*) glycerin, and 0.5% (*w*/*v*) polyvinylpolypyrrolidone, and were supplemented with protease inhibitors (cOmplete ULTRA Tablets, Merck, Darmstadt, Germany). The samples were then homogenized with a homogenizer (AM-3; Nippon Seiki Seisakusho, Tokyo, Japan) at 10,000 rpm for 70 s. The mixture was passed through doubled Miracloth (Merck) to obtain a filtrate. After centrifugation (7000× *g*, 20 min, 4 °C) to remove tissue debris, the resulting supernatant was ultracentrifuged (100,000× *g*, 30 min, 4 °C; OptimaXE-90; Type 70.1 Ti; Beckman Coulter, Brea, CA, USA) to collect microsomal fractions. The supernatant was discarded, and the pellet was suspended in resuspension buffer, which included 10 mM HEPES–KOH (pH 6.8), 1 mM EDTA, and 10% glycerin, and subjected to ultracentrifugation again. After suspending and dispensing using a potter-type Teflon homogenizer, the microsomal membrane fraction was rapidly frozen using liquid nitrogen and stored at −80 °C.

### 4.6. Transport Experiments

The incubation mixture was prepared by adding a membrane fraction to a solution of 5 mM ATP/MgSO_4_ plus 50 mM HEPES-KOH buffer (pH 7.5) supplemented with 100 µM of various lignin precursors (Figure 3) as the substrate to give a protein content of approximately 10 µg/100 µL, which was then incubated at 28 °C for 10 min. Then, the incubation mixture (80 µL) was gel-filtered through a spin column packed with Sephadex G50, and then 20 µL of methanol was added to 60 µL of filtrate. After centrifugation (20,000× *g*, 15 min, 4 °C), the supernatant was passed through a filter (DISMIC-03CP 0.45 μm; ADVANTEC, Tokyo, Japan) to remove the precipitate and then subjected to an HPLC analysis as previously described [20].

## Figures and Tables

**Table 1:** Samples used in the present study.

Sample Name	Sampling Date	Total Culm Length (m)
M-Apr	17 April 2018	1.91 ± 0.08
L-Apr	30 April 2018	5.63 ± 0.27
May	15 May 2018	15.95 ± 1.10
Jun	20 June 2017	15.94 ± 1.35
Jul	28 July 2017	16.84 ± 0.60
Aug	27 August 2017	15.10 ± 1.78
L-Apr (2020)	30 April 2020	5.17 ± 0.61

To obtain samples at a uniform developmental stage in each sampling, three culms (a–c) of similar lengths were collected. Length data are means ± SDs of three biological replicates. M, mid; L, late.

## Data Availability

All data generated or analyzed during this study are included in this published article and Supplementary Information files.

## Supplementary Materials

The following are available online at https://www.mdpi.com/article/10.3390/plants10112237/s1, Figure S1: Measurement of ATPase activity in microsomal membrane; Figure S2: Fractionation of crude microsomal membranes.
