# Supplementary material for: Active Transport of Lignin Precursors into Membrane Vesicles from Lignifying Tissues of Bamboo
Source: Plants (Basel). 2021 Oct 20;10(11):2237. doi: 10.3390/plants10112237 (PMC8620782; doi:10.3390/plants10112237)
Supplement: Supplementary file 1 [file plants-10-02237-s001.zip › plants-1383229-supdone.pdf]

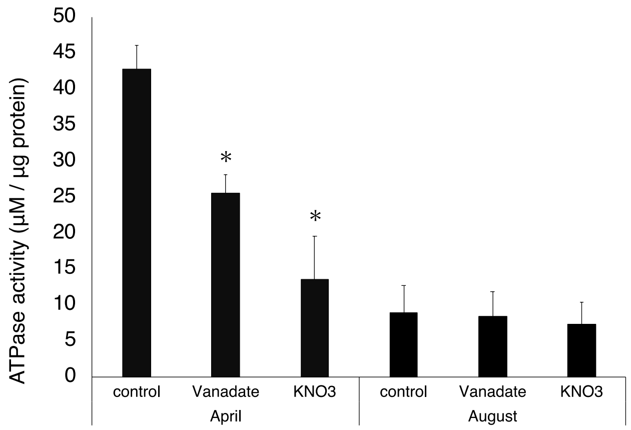

Figure S1. Measurement of ATPase activity in microsomal membrane fractions. The samples used for determining H^+-ATPase activity were collected in M-Apr and August (see Table 1). The activity values were obtained after addition of 1 mM vanadate or 50 mM KNO_3. Control represents activity without an inhibitor. Data are the means of three replicates (error bars = SDs). *$p < 0.05$ (Dunnett test).

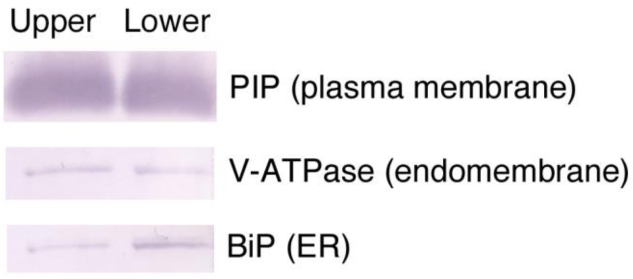

Figure S2. Fractionation of crude microsomal membranes. Crude microsomal membranes of moso bamboo were partitioned by aqueous two-polymer phase system (Yoshida et al., 1983). Plasma membrane intrinsic protein (PIP), V-ATPase, and BiP were immunodetected in each fraction. Upper; upper phase, Lower; lower phase of the aqueous two-polymer system.

Supplemental reference

Yoshida S, Uemura M, Niki T, Sakai A, Gusta LV. Partition of membrane particles in aqueous two-polymer phase system and its practical use for purification of plasma membranes from plants. *Plant Physiol*. 1983, 72, 105-114.
